# Liver Transplantation for Hepatocellular Carcinoma in the Era of Immune Checkpoint Inhibitors

**DOI:** 10.3390/cancers16132374

**Published:** 2024-06-28

**Authors:** Nicola De Stefano, Damiano Patrono, Fabio Colli, Giorgia Rizza, Gianluca Paraluppi, Renato Romagnoli

**Affiliations:** General Surgery 2U-Liver Transplant Unit, Azienda Ospedaliero Universitaria Città della Salute e della Scienza di Torino, University of Torino, Corso Bramante 88-90, 10126 Torino, Italy; n.destefano@unito.it (N.D.S.); damiano.patrono@unito.it (D.P.); fabiocolli86@gmail.com (F.C.); giorgia.rizza@gmail.com (G.R.); gparaluppi68@gmail.com (G.P.)

**Keywords:** liver transplantation, immunotherapy, immune checkpoint inhibitors, hepatocellular carcinoma, acute rejection

## Abstract

**Simple Summary:**

Immune checkpoint inhibitors (ICIs) have witnessed significant success in systemic therapy of hepatocellular carcinoma, stimulating the transplant community to consider their use in the liver transplantation (LT) setting. This review provides an updated overview of the current evidence on ICI therapy before and after LT, discussing the associated pitfalls and remaining challenges. A particular focus is placed on the interactions between ICIs and immunosuppressive drugs, in order to identify predictive factors of good response, as well as critical aspects that should guide future research on the topic.

**Abstract:**

Hepatocellular carcinoma (HCC) remains the leading oncological indication for liver transplantation (LT), with evolving and broadened inclusion criteria. Immune checkpoint inhibitors (ICIs) gained a central role in systemic HCC treatment and showed potential in the peri-transplant setting as downstaging/bridging therapy before LT or as a treatment for HCC recurrence following LT. However, the antagonistic mechanisms of action between ICIs and immunosuppressive drugs pose significant challenges, particularly regarding the risk of acute rejection (AR). This review analyzes the main signaling pathways targeted by ICI therapies and summarizes current studies on ICI therapy before and after LT. The literature on this topic is limited and highly heterogeneous, precluding definitive evidence-based conclusions. The use of ICIs before LT appears promising, provided that a sufficient wash-out period is implemented. In contrast, the results of post-LT ICI therapy do not support its wide clinical application due to high AR rates and overall poor response to treatment. In the future, modern graft preservation techniques might support the selection of good ICI responders, but data from high-level studies are urgently needed.

## 1. Introduction

Hepatocellular carcinoma (HCC) is the most prevalent oncological indication for liver transplantation (LT). While the Milan criteria established by Mazzaferro et al. [1] continue to serve as the gold standard, more inclusive criteria have emerged over the past two decades to expand LT access to a growing number of patients, while ensuring their best chance of survival [2,3,4]. This has led to a complex decision-making landscape, further influenced by the increasing acceptance of extended criteria donors (ECD) and significant advancements in downstaging techniques [4]. The Barcelona Clinic Liver Cancer (BCLC) guidelines are widely used to inform the management of HCC. Notably, their latest 2022 update included extended LT criteria and downstaging techniques into the decision-making algorithm, making LT an option for HCC patients previously deemed ineligible [5].

Another significant advancement in HCC treatment, as emphasized by the BCLC guidelines, pertains to systemic therapy [5]. New drug combinations have surpassed Sorafenib as the first-line treatment for intermediate and advanced HCC stages, owing to an enhanced understanding of HCC biological behavior. Specifically, immune checkpoint inhibitors (ICIs) are poised to become the cornerstone of systemic HCC treatment [6].

More recently, ICIs have also been administered in the peri-transplant setting, both for tumor downstaging and for treating HCC recurrence following LT. However, it is important to note that there is often an overlapping—and antagonistic—mechanism of action between ICIs and immunosuppressive drugs, while modulation of the immune response remains an inevitable necessity for LT patients. As the use of ICIs in the peri-transplant setting is still subject to debate, this paper aims to elucidate the most relevant mechanisms of action of the different molecules and to provide an up-to-date literature review on their use before and after LT.

## 2. Materials and Methods

The Medline (PubMed) database was accessed on 29 February 2024 and searched for ‘HCC’ AND ‘immunotherapy’ AND ‘transplant*’, retrieving 743 articles. The literature review was performed by 2 authors (DP and NDS) and any disagreement was resolved by consensus. Titles were screened to select potentially relevant studies, initially including 225 articles. Next, abstracts of the selected items were screened according to the inclusion and exclusion criteria, leading to 57 articles being eligible for full-text review. A total of 20 additional articles were identified by manual cross-checking among the cited references and 2 recently published papers were added during the revision process, resulting in 79 included articles. Inclusion criteria were clinical and preclinical peer reviewed studies reporting on the use of ICIs in the LT with no species, age, or sex restriction. Publications with no full text available or published in languages other than English were excluded.

## 3. Results

### 3.1. ICIs for Advanced HCC

#### 3.1.1. PD-1 and PD-L1

Programmed Cell Death Protein 1 (PD-1) inhibits immune responses and fosters self-tolerance by regulating T-cell activity, triggering apoptosis in antigen-specific T cells, and preventing apoptosis in regulatory T cells [7]. Programmed Cell Death Ligand 1 (PD-L1) is a trans-membrane protein acting as a co-inhibitory factor in immune responses. PD-L1 binds with PD-1, reducing the proliferation of PD-1 positive cells, suppressing their cytokine secretion, and inducing apoptosis. Under physiological conditions, the PD-1/PD-L1 axis holds a pivotal role in maintaining peripheral tolerance. However, in several diseases, the activation of the PD-1/PD-L1 signaling has the potential to inhibit immune cell activation. This mechanism is often exploited by tumor cells to evade antitumor immune surveillance.

Several monoclonal antibodies have been developed to selectively bind PD-1, such as Nivolumab and Pembrolizumab, or PD-L1, such as Atezolizumab and Durvalumab. The Checkmate 040 was the first landmark study demonstrating safety and efficacy of Nivolumab for HCC patients not responding to prior treatments, including Sorafenib [8]. The trial reported an objective response rate (ORR) of 20% and an overall survival (OS) of 15 months. However, the subsequent Checkmate 459 comparator study did not reveal a survival benefit of first-line Nivolumab compared to Sorafenib [9]. Nevertheless, the experimental group exhibited a higher ORR (15% vs. 7%) and a lower incidence of grade 3/4 adverse events (22% vs. 49%). The Keynote 240 trial evaluated Pembrolizumab as a second-line treatment in advanced HCC, demonstrating a 36-month progression-free survival (PFS) rate of 8.9%, compared to 0% for placebo, and an ORR of 18.3%, compared to 4.4% for placebo [10]. Studies involving PD-L1 inhibitors have also shown promising results. The Imbrave 050 trial compared the combination of Atezolizumab and Bevacizumab, an antiangiogenic monoclonal antibody, with Sorafenib in advanced HCC [11]. The experimental group exhibited one-year OS of 67% and median PFS of 6.8 months, compared to 55% and 4.3 months, respectively, with Sorafenib. Based on these favorable findings, the combination of Atezolizumab/Bevacizumab is now recommended by the BCLC guidelines as first-line therapy for patients with advanced HCC [5].

#### 3.1.2. CTLA-4

Cytotoxic T-lymphocyte-associated antigen 4 (CTLA-4) is a protein receptor that functions as an immune checkpoint by downregulating T-cell responses [7,12]. It is expressed by activated T cells and regulatory T cells. CTLA-4 shares homology with CD28 and competes with it for binding to CD80 (B7-1) and CD86 (B7-2) on antigen-presenting cells (APCs). Despite sharing ligands with CD28, CTLA-4 exhibits higher affinity and avidity for CD80 and CD86, enabling it to outcompete CD28 and transmit an inhibitory signal to T cells, in contrast to the stimulatory signal transmitted by CD28. Similarly to the PD-1 pathway, the overexpression of CTLA-4 is a hallmark of T-cell exhaustion, as it occurs during chronic infections and in the cancer microenvironment, resulting in a dampening of the immune system.

Tremelimumab is a monoclonal antibody directed against CTLA-4 that showed safety and manageable toxicity in advanced HCC patients not responding or intolerant to Sorafenib, while achieving a median OS of 15 months [13]. However, CTLA-4 inhibitors have been less frequently utilized as monotherapy, as the best results have been achieved when these ICIs are used in combination with other molecules.

#### 3.1.3. PD-1 and CTLA-4 Synergic Inhibition

Both CTLA-4 and PD-1 pathways exert similar negative effects on T-cell activity but vary in their mechanistic activity [14]. Unlike PD-1, which is predominantly activated during the effector phase of T-cell response, CTLA-4 functions during the priming phase of T-cell activation. Moreover, while CTLA-4 is expressed on T cells only, PD-1 is present on B cells and myeloid cells too. It was postulated that simultaneous blockades of both pathways could lead to improved efficacy over CTLA-4 or PD-1 inhibition alone.

The combination of Tremelimumab and Durvalumab achieved the highest OS and ORR (18.7 and 24%, respectively) when compared to their use as single agents in advanced HCC patients, making this protocol a valid option in patients who progress on, are intolerant to, or refuse Sorafenib [13]. In the Checkmate 040 trial, HCC patients not responding to Sorafenib were treated with the combination of Nivolumab and Ipilimumab, another CTLA-4 inhibitor, at three different dosages [15]. The higher dose was correlated with the longest OS (23 months) and the highest ORR (32%), albeit with adverse events observed in nearly every patient of the arm. The recent landmark Himalaya trial investigated the combination of Tremelimumab and Durvalumab for unresectable HCC, showing increased OS at 36 months than Durvalumab alone or Sorafenib (30.7%, 24.7%, and 20.2%, respectively) [16]. Recently published long-term follow-up data revealed 48-month OS of 25% in the Tremelimumab plus Durvalumab group and 15% in the Sorafenib group [17], supporting this new combination as first-line treatment in advanced HCC [5].

### 3.2. ICIs and LT

The significant success of ICIs in advanced HCC has stimulated interest in their use even in less advanced stages, where resective or ablative treatments guarantee optimal survival but are affected by consistently high recurrence rates, reportedly higher than 70% within 5 years, even in patients considered good candidates for curative treatment [18]. The Imbrave 050 trial was the first successful study comparing adjuvant ICI therapy to active surveillance in patients with high-risk resected or ablated HCC [18]. The combination of Atezolizumab and Bevacizumab was associated with significantly reduced recurrence-free survival (RFS), prompting further studies on ICIs in the perioperative setting.

**Figure 1:**
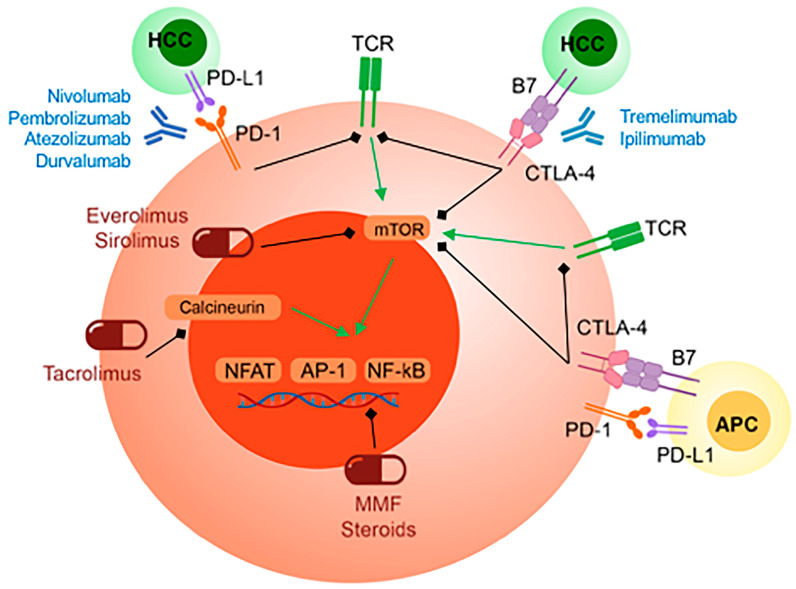
Overlapping pathways between ICIs and immunosuppressive drugs on T-cell activation. Both PD-1/PD-L1 and CTLA-4 blockades by ICIs result in activation of TCR and mTOR signaling, eventually upregulating NFAT, NF-kB, and AP-1 genes to further promote T-cell proliferation and cytokine expression. In contrast, immunosuppressive drugs, either targeting mTOR or directly interfering with the expression of the aforementioned genes, result in suppression of T-cell activation.

However, the scenario slightly changes when considering the use of ICIs in the peri-transplant setting. Indeed, ICIs and immunosuppressive drugs target the same biological pathways exploiting opposite mechanisms. Specifically, all classes of immunosuppressive drugs currently utilized in LT, including calcineurin inhibitors (CNIs), mammalian target of rapamycin (mTOR) inhibitors, mycophenolate mofetil, and steroids, inhibit the expression of NFAT, NF-kB, and AP-1 genes, ultimately resulting in PD-1 and CTLA4 downregulation and T-cell inactivation (Figure 1). Thus, the use of ICIs in LT patients was approached with caution, as they may reduce the efficacy of immunosuppression, potentially leading to allograft rejection (AR). Nevertheless, a growing number of studies have been published in recent years reporting early optimistic data.

#### 3.2.1. ICIs before LT

Similarly to locoregional therapies, ICIs can be used as neoadjuvant downstaging or bridging therapy in HCC patients not meeting transplant criteria. However, there is an anticipated increased risk of AR if ICIs are administered before LT. This concern has limited the development of shared protocols, and several centers have applied this approach in isolated cases or small case series heterogeneously. Table 1 summarizes all case reports and case series reporting on ICI therapy prior to LT. Although comparisons are challenging due to the heterogeneity of ICI protocols, clinical HCC features, and time-to-transplant, it is evident that favorable outcomes and reasonable safety can be achieved.

Nivolumab, either as a monotherapy or in combination with other agents, was the most frequently utilized ICIs (13 studies) [19,20,21,22,23,24,27,29,30,32,34,35,37], followed by Atezolizumab combined with Bevacizumab or Nivolumab (four studies) [28,33,34,36]. All protocols included a PD-1/PD-L1 inhibitor, whereas CTLA-4 inhibitors were considered in only two cases, both in combination with Nivolumab [23,34]. Nineteen (30.6%) out of 62 patients experienced AR. Most AR cases were effectively managed with steroid pulses and/or adjustment of the immunosuppressive regimen; however, graft loss was reported for four cases [19,22,30]. Notably, all patients who experienced AR had received their last dose of ICIs less than 90 days before LT.

Nordness et al. [19] were the first to report massive necrosis of the transplanted liver following neoadjuvant Nivolumab therapy, eventually resulting in patient death. Subsequently, four other cases were reported by two centers, all requiring urgent re-LT due to AR [19,22,30]. This heightened attention to the wash-out period between the last Nivolumab dose and LT. When this period exceeded 2 months, no cases of AR were reported. Surprisingly, Tabrizian et al. [27] conducted nine LTs following downstaging with Nivolumab, and no cases of severe AR were observed, even with wash-out periods as short as one day. However, the same group recently presented the outcomes of their multicenter prospective study, revealing that a wash-out period shorter than 90 days was associated with an increased risk of AR [38]. Data from other classes of ICIs are particularly scarce, making it difficult to draw any conclusion. However, it is worth noting that when ICIs were administered in combination instead of monotherapy, no AR was observed except for one case that was successfully managed with steroid pulses [21,23,25,28,33,36].

Recently, Guo et al. [39] presented the results of a multicenter cohort study investigating the safety of pre-LT ICI therapy. The study included eighty-three LTs, totalizing the largest cohort available to date. ICI therapies utilized in the study comprised Camrelizumab, Pembrolizumab, Sintilimab, Tislelizumab, Nivolumab, and Atezolizumab. Interestingly, in contrast to previous literature, the latter two were the least utilized, with Nivolumab administered to only five patients and Atezolizumab to four. Notably, AR occurred in nearly 28% of recipients, with six cases resulting in AR-related deaths. Multivariate analysis identified a wash-out period of at least 30 days as the only significant protective factor against AR. These findings further advocate for proceeding with caution and carefully considering a wash-out period of at least three half-lives before proceeding with LT (Table 2).

**Figure 2:**
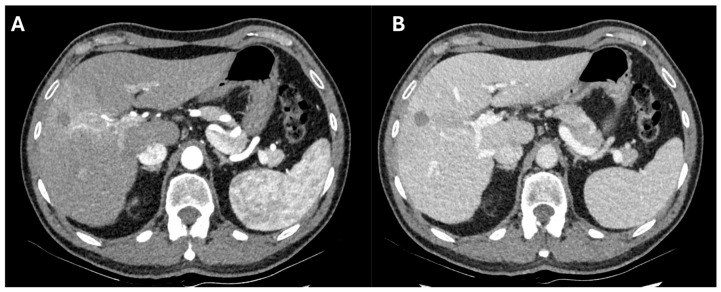
Computed tomography arterial (**A**) and portal (**B**) phase of a case of successful downstaging by a combination of loco-regional therapies and immunotherapy. After a microwave ablation of a Sg8 HCC, the patient developed a neoplastic thrombosis of Sg8 Glissonean pedicle. He was treated with trans-arterial radio-embolization followed by 20 cycles of Atezolizumab–Bevacizumab. After disappearance of contrast-enhancing tissue at the Sg8 pedicle and normalization of alpha-fetoprotein (from 42.9 to 8.1 ng/mL), the patient was waitlisted for liver transplantation 1 month after the last administration of immunotherapy. He was transplanted 10 days after and had an uneventful postoperative course, with no sign of acute rejection. At 5-month follow-up, he had normal liver function and no evidence of recurrence.

On the other hand, it could be speculated that delaying LT too long after the last dose of ICI might increase the risk of HCC recurrence, further complicating the decision-making process in terms of timing balance. Moreover, this timing is often unpredictable given the scarcity of available donors. HCC recurrence rates in the two largest series on pre-LT ICIs were markedly different. Tabrizian et al. [38] observed HCC recurrence in 4% of recipients at a median time of 24 months, while Guo et al. [39] reported HCC recurrence in 24% of recipients at a median time of 5.5 months. The reasons for these differences should be sought in the extreme heterogeneity of the listing protocols of the studies, whether ICIs were used as bridging or downstaging therapy, whether patients underwent additional treatments other than ICIs, and in relation to the tumor burden at the time of LT. Moreover, the oncological efficacy of ICIs might be improved if combined with locoregional therapies, which might reduce the number of cycles needed to achieve LT criteria (Figure 2). Currently, five trials are investigating this approach (NCT05185505, NCT05339581, NCT05717738, NCT03817736, NCT05475613).

#### 3.2.2. ICI after LT

Immunosuppression favors the development of de novo malignancies or tumor recurrence, making the onset of tumors one of the leading causes of mortality in LT patients. Moreover, with the number of LTs for oncological indications projected to rise [40], novel strategies to manage tumor recurrence are urgently needed. The application of adjuvant protocols in cases of LT for HCC with a high risk of recurrence has been poorly investigated and with inconclusive results [41], leaving the decision to start a treatment only after the diagnosis of malignancy is made. In this scenario, prognosis is heavily dependent on the feasibility of surgical/locoregional approaches, as the efficacy of systemic treatments is generally dismal [42]. ICIs in this setting appear to be even more risky, given the need for concomitant immunosuppressive therapy. Table 3 summarizes case reports and case series reporting ICI therapy following LT.

A total of 32 studies have been published on ICIs in LT patients, of which 24 are case reports, confirming the low level of evidence on this topic [35,43,44,45,46,47,48,49,50,51,52,53,54,55,56,57,58,59,60,61,62,63,64,65,66,67,68,69,70,71,72,73]. In cases of HCC recurrence, ICIs were used as second- or third-line therapies following locoregional and systemic treatments. The most commonly used ICI was Nivolumab. In most cases, immunosuppression was tapered to maintain a single drug regimen concurrently with ICI therapy, aiming to limit pharmacological interference while minimizing the risk of AR. However, even considering the limitation of drawing conclusions from highly heterogeneous cases, the overall results were quite disappointing, with an acute AR rate of 27% and a response to ICIs, defined as at least disease stability during treatment, of less than 33%. Three aspects clearly need to be elucidated to optimize this therapeutic approach: identifying the right timing, the appropriate immunosuppressive regimen, and potential responders.

In cases of HCC recurrence (Table 4), the median time from LT to recurrence was 24 months, with a systemic presentation observed in 17 out of 21 patients. Interestingly, no cases of AR were reported if recurrence occurred later than 3 years from LT [48,53,54,62,63,65], as well as if recurrence involved the liver only [58,63,68]. This observation could identify a subgroup of patients in whom the longer period from LT guarantees a lower risk of AR on one hand, while on the other hand, the liver-only pattern suggests a less aggressive tumor behavior, potentially more amenable to curative ICI therapy. 

In their single-institution series, Abdel-Wahab et al. [56] observed that single-agent CNI immunosuppression was associated with the lowest AR rate (11%). Quite surprisingly, only one preclinical animal study has investigated post-LT ICI to date. Hsu et al. [74] demonstrated in murine models of allogeneic skin transplantation and syngeneic subcutaneous and orthotopic HCC models that the combination of high-dose Tacrolimus and PD-1 blockade was able to reduce tumor growth while preventing AR. They suggested that the antitumor effect was strongly supported by natural killer (NK) cells, on which the PD-1 blockade had greater efficacy than CNI inhibition. This observation was further supported by a recent Japanese clinical trial in which adaptive immunotherapy using donor-liver-derived NK was administrated to 38 living-donor-LT patients to prevent HCC recurrence [75]. After a median follow-up of 8 years, recipients treated with NK cells had significantly higher 1-year OS (97.4% vs. 81.8%), and those stratified with intermediate risk of recurrence showed the highest treatment benefit in terms of recurrence rates. Investigating the molecular basis behind ICIs and immunosuppressive drugs would provide a more solid foundation to choose the optimal combination for the clinical setting, and additional evidence is strongly warranted.

Finally, to improve patient selection and mitigate unnecessary adverse events or tumor progression, the identification of predictive biomarkers is imperative. Shi et al. [68] advocated for assessing liver graft PD-L1 expression prior to ICI therapy, as they found that a negative PD-L1 profile was never associated with AR. Conversely, high PD-L1 expression was correlated with a favorable response to Pembrolizumab in a subset of patients in the Keynote 244 trial [76]. These findings are, once again, not surprising given the dual nature of the PD-1/PD-L1 axis in this context. Studies on gene expression profiling are eagerly awaited to discover novel biomarkers and develop a personalized immunotherapy regimen tailored to individual response probabilities and risk of AR.

## 4. Conclusions and Future Perspectives

ICI therapy has rapidly gained success in the landscape of advanced HCC, prompting the transplant community to investigate their efficacy in the peri-transplant setting, despite a degree of skepticism regarding potential pharmacodynamic interactions between ICIs and immunosuppressive agents. No definitive conclusion can be drawn given the inherent limitations of the available literature, including the high heterogeneity of administered protocols, low numbers, and the lack of biomolecular studies investigating the susceptibility of treated patients to develop AR and of treated tumors to respond to therapy. Furthermore, a certain degree of publication bias cannot be excluded. Thus, it is possible that the applicability and efficacy of ICI-based therapies is overestimated by the available literature and that larger studies will provide a more balanced view. With these limitations it can be recommended that, when ICI are administered before LT, a mandatory wash-out period should be expected before listing the patient to reduce the risk of AR. Current evidence, albeit derived from low-level studies, suggests that this period should be at least three half-lives of the administered ICI. Notwithstanding the need to define the right timing, neoadjuvant ICI therapy will likely become a viable option in the armamentarium of therapeutic strategies to ensure access to LT for HCC patients.

In contrast, the results of post-LT ICI therapy have been more disappointing, with an AR rate that currently makes this approach difficult to justify on a large scale, given the limited therapeutic benefit. However, the growing utilization of ECD grafts, the increasing adoption of Milan-out criteria, and the expanding range of oncological indications for LT will make it increasingly necessary to deal with post-LT tumor recurrence in the near future. In this context, the introduction of machine perfusion (MP) technology could be a valuable ally in two ways. On one hand, the reduction in IRI offered by MP appears to reduce the incidence of HCC recurrence [77], although this hypothesis is still to be validated [78]. On the other hand, the development of long-term MP [79] could offer an ideal time window to perform biomolecular tests on the graft, such as PD-L1 expression [68], to base the allocation on anticipated probabilities of AR. Preclinical studies to clarify the molecular mechanisms underlying AR in the context of ICI therapy and, thus, identify such response biomarkers are urgently needed, along with shared registries and multicenter studies to address the current lack and dispersion of clinical data on the topic.

## Figures and Tables

**Table 1:** Studies on ICIs as neoadjuvant therapies before LT for HCC.

Study	Type (n)	ICI	Dose	Duration	Wash-Out	IS Regimen	Rejection	Treatment
Nordness et al., 2019 [19]	Case report (1)	Nivolumab	240 mg every 2 weeks	19 months	8 days	Tac, MMF, Steroids	Yes	Steroids, Thymo (patient died)
Schwacha-Eipper et al., 2020 [20]	Case report (1)	Nivolumab	NR	34 cycles	15 weeks	NR	No	-
Chen et al., 2021 [21]	Case series (5)	Nivolumab	3 mg/kg every 2 weeks	NR	7 days	Tac, MMF	No	-
					16 weeks		No	-
					8 weeks		No	-
					8 weeks		No	-
					8 weeks		No	-
Dehghan et al., 2021 [22]	Case report (1)	Nivolumab	240 mg every 2 weeks	1 month	5 weeks	Tac, MMF, Steroids	Yes	Steroids, Thymo, Plasma exchange, Re-LT
			480 mg every 4 weeks	15 months				
Lizaola-Mayo et al., 2021 [23]	Case report (1)	Nivolumab Ipilimumab	NR	6 months	9 weeks	Basiliximab + Steroids [induction], Tac, MMF, Steroids	No	-
Peterson et al., 2021 [24]	Case report (1)	Nivolumab	240 mg every 2 weeks	6 months	40 weeks	NA	No	-
Qiao et al., 2021 [25]	Case series (7)	Pembrolizumab + Lenvatinib	200 mg, 3 weeks per cycle	3 cycles (average)	6 weeks (average)	Basiliximab + Steroids [induction], Tac, MMF, Sirolimus, Steroids	1 case	Steroids
		Camrelizumab + Lenvatinib	200 mg, 2 weeks per cycle					
Sogbe et al., 2021 [26]	Case report (1)	Durvalumab	NR	20 months	12 weeks	Tac, MMF, Steroids	No	-
Tabrizian et al., 2021 [27]	Case series (9)	Nivolumab	240 mg every 2 weeks	21 cycles	18 days	Tac, MMF, Steroids	No	-
				8 cycles	3 weeks		No	-
				32 cycles	1 day		No	-
				4 cycles	2 days		No	-
				25 cycles	3 weeks		Yes	Increase Tac
				4 cycles	2 weeks		No	-
				9 cycles	36 weeks		No	-
				12 cycles	7 days		No	-
				2 cycles	4 weeks		No	-
Abdelrahim et al., 2022 [28]	Case report (1)	Atezolizumab	1200 mg	6 cycles	8 weeks	Tac, MMF	No	-
		Bevacizumab	15 mg/kg	5 cycles				
Aby et al., 2022 [29]	Case report (1)	Nivolumab	480 mg every 4 weeks	23 cycles	16 days	Tac, MMF, Steroids	Yes	Steroids, Thymo
Dave et al., 2022 [30]	Case series (5)	Nivolumab	NR	NR	15 weeks (average)	NR	2 cases	Re-LT
Kang et al., 2022 [31]	Case report (1)	Pembrolizumab	2 mg/kg every 3 weeks	3 cycles	20 weeks	NR	No	-
Schnickel et al., 2022 [32]	Case series (5)	Nivolumab	240 mg every 2 weeks twice then 480 mg every 4 weeks	18 months	5 weeks	Tac, MMF, Steroids	Yes	Steroids, Thymo, Plasma exchange
				8 months	10 days		Yes	Steroids, Thymo, Rituximab
				8 months	12 weeks		No	-
				12 months	16 weeks		No	-
				12 months	24 weeks		No	-
Chouik et al., 2023 [33]	Case report (1)	Atezolizumab	1200 mg	18 cycles	7 days	Basiliximab + Steroids [induction], Tac, MMF, Steroids	No	-
		Bevacizumab	15 mg/kg					
Ohm et al., 2023 [34]	Case series (3)	Atezolizumab + Nivolumab	1200 mg + 15 mg/kg every 3 weeks	7 cycles	33 weeks	NR	No	-
		Ipilimumab + Nivolumab (then Nivolumab alone)	3 mg/kg + 1 mg/kg every 3 weeks followed by 480 mg every 4 weeks	4 + 3 cycles	2 days		No	-
		Atezolizumab + Nivolumab	1200 mg + 15 mg/kg every 3 weeks	6 cycles	7 days		No	-
Rudolph et al., 2023 [35]	Case report (1)	Nivolumab	NR	7 cycles	1 week	Tac, MMF, Steroids	GVHD	Steroids, Thymo
Schmiderer et al., 2023 [36]	Case report (1)	Atezolizumab	1200 mg	6 months	6 weeks	Tac, MMF, Steroids	No	-
		Bevacizumab	15 mg/kg	6 months				
		Lenvatinib (started at listing)	8 mg	6 weeks				
Wang et al., 2023 [37]	Case series (16)	Nivolumab	3 mg/kg every 2 weeks	6 cycles	3 weeks	Basiliximab + Steroids [induction], Tac, Sirolimus, Steroids	No	-
		Nivolumab	3 mg/kg every 2 weeks	4 cycles	9 weeks		No	-
		Pembrolizumab	200 mg every 3 weeks	4 cycles	24 weeks		No	-
		Pembrolizumab	200 mg every 3 weeks	2 cycles	3 weeks		Yes	Steroids/Increase Tac
		Pembrolizumab	200 mg every 3 weeks	4 cycles	6 weeks		No	-
		Pembrolizumab	200 mg every 3 weeks	1 cycle	4 weeks		Yes	Steroids/Increase Tac
		Pembrolizumab	200 mg every 3 weeks	3 cycles	3 weeks		Yes	Steroids/Increase Tac
		Pembrolizumab	200 mg every 3 weeks	3 cycles	7 days		Yes	Steroids/Increase Tac
		Pembrolizumab	200 mg every 3 weeks	3 cycles	2 weeks		Yes	Steroids/Increase Tac
		Sintilimab	200 mg every 3 weeks	2 cycles	5 weeks		No	-
		Sintilimab	200 mg every 3 weeks	4 cycles	4 weeks		Yes	Steroids/Increase Tac
		Sintilimab	200 mg every 3 weeks	8 cycles	2 weeks		Yes	Steroids/Increase Tac
		Sintilimab	200 mg every 3 weeks	10 cycles	3 weeks		Yes	Steroids/Increase Tac
		Camrelizumab	3 mg/kg every 3 weeks	5 cycles	13 weeks		Yes	Steroids/Increase Tac
		Camrelizumab	3 mg/kg every 3 weeks	6 cycles	26 weeks		No	-
		Nivolumab Toripalimab Sintilimab Tislelizumab	200 mg every 2/3 weeks	27 cycles	10 weeks		No	-

Abbreviations: GVHD, graft-versus-host disease; ICIs, immune checkpoint inhibitors; IS, immunosuppression; MMF, mycophenolate mofetil; NR, not reported; LT, liver transplantation; Tac, Tacrolimus; Thymo, Thymoglobulin.

**Table 2:** Half-life and molecular target of ICIs administered prior to LT.

ICI	Half-Life (Days)	Target
Nivolumab	25.0	PD-1
Pembrolizumab	22.0	PD-1
Camrelizumab	5.5	PD-1
Sintilimab	19.6	PD-1
Tislelizumab	13.3	PD-1
Toripalimab	12.6	PD-1
Atezolizumab	27.0	PD-L1
Durvalumab	18.0	PD-L1
Ipilimumab	14.7	CTLA-4

Abbreviations: CTLA-4, cytotoxic T-lymphocyte-associated antigen-4, ICI, immune checkpoint inhibitor; PD-1, programmed cell death-1; PD-L1, programmed cell death-ligand 1.

**Table 3:** Studies on ICI therapy after LT.

Study	Type [n]	ICI	Indication	Duration	Time from LT	IS Regimen	Rejection	ICI Response
Morales et al., 2015 [43]	Case report (1)	Ipilimumab	Melanoma	4 cycles	8 years	Tac	No	Yes
Ranganath et al., 2015 [44]	Case report (1)	Ipilimumab	Melanoma	4 cycles	8 years	Tac	No	No
De Toni et al., 2017 [45]	Case report (1)	Nivolumab	HCC recurrence	15 cycles	11 years	Tac	No	No
Dueland et al., 2017 [46]	Case report (1)	Ipilimumab	Melanoma	1 cycle	1.5 years	Steroid	Yes	-
Friend et al., 2017 [47]	Case series (2)	Nivolumab	HCC recurrence	2 cycles	3 years	Sirolimus	Yes	-
				1 cycle	4 years	Tac	Yes	
Varkaris et al., 2017 [48]	Case report (1)	Pembrolizumab	HCC recurrence	4 months	8 years	Tac	No	No
Biondani et al., 2018 [49]	Case report (1)	Nivolumab	NSCLC	3 cycles	13 years	Tac, Everolimus, Steroid	No	No
DeLeon et al., 2018 [50]	Case series (7)	Nivolumab	HCC recurrence	1.2 months	2.7 years	Tac	No	No
		Nivolumab	HCC recurrence	1.1 months	7.8 years	MMF, Sirolimus	No	No
		Nivolumab	HCC recurrence	1.3 months	3.7 years	Tac	No	No
		Nivolumab	HCC recurrence	0.3 months	1.2 years	Tac	No	No
		Nivolumab	HCC recurrence	0.9 months	1.1 years	Sirolimus	Yes	-
		Pembrolizumab	Melanoma	9.5 months	5.5 years	MMF, Everolimus	No	Yes
		Pembrolizumab	Melanoma	0.7 months	1.1 years	MMF, Steroid	Yes	-
Gassmann et al., 2018 [51]	Case report (1)	Nivolumab	HCC recurrence	1 cycle	2 years	MMF, Everolimus	Yes	-
Kuo et al., 2018 [52]	Case report (1)	Ipilimumab followed by Pembrolizumab	Melanoma	4 cycles + 25 cycles	1 years	Sirolimus	No	Yes
Nasr et al., 2018 [53]	Case report (1)	Pembrolizumab	HCC recurrence	NR	4 years	Tac, MMF	No	Yes
Rammohan et al., 2018 [54]	Case report (1)	Pembrolizumab	HCC recurrence	10 months	3 years	Tac	No	Yes
Tio et al., 2018 [55]	Case report (1)	Pembrolizumab	Melanoma	NR	NR	Cyclosporine	Yes	-
Abdel-Wahab et al., 2019 [56]	Case series (11)	Ipilimumab	Melanoma	NR	1.5 years	Steroid	Yes	-
		Pembrolizumab	Melanoma	NR	5 years	Sirolimus	Yes	-
		Nivolumab	HCC recurrence	NR	3.3 years	Sirolimus	Yes	-
		Nivolumab	HCC recurrence	NR	1.9 years	Tac	Yes	-
		Ipilimumab	Melanoma	NR	8 years	Sirolimus	No	Yes
		Pembrolizumab	Melanoma	NR	20 years	Tac	No	Yes
		Ipilimumab	Melanoma	NR	8 years	Tac	No	No
		Nivolumab	HCC recurrence	NR	0.92 years	Tac	No	No
		Pembrolizumab	HCC recurrence	NR	8 years	Tac	No	No
		Pembrolizumab	Melanoma	NR	6 years	MMF, Sirolimus	No	Yes
		Nivolumab	NSCLC	NR	13 years	Steroid, Tac, Everolimus	No	No
Chen et al., 2019 [21]	Case report (1)	Pembrolizumab	Metastatic CRC	15 cycles	4 years	Tac, Steroid	No	Yes
Lee et al., 2019 [57]	Case report (1)	Nivolumab	SCC	2 cycles	1 year	Everolimus	Yes	-
Al Jarroudi et al., 2020 [58]	Case series (3)	Nivolumab	HCC recurrence	4 cycles	3 years	Tac	No	No
				5 cycles	1 year		No	No
				6 cycles	5 years		No	No
Amjad et al., 2020 [59]	Case report (1)	Nivolumab	HCC recurrence	20 months	2 years	Tac, MMF, Steroid	No	Yes
Anugwom et al., 2020 [60]	Case report (1)	Nivolumab	HCC recurrence + NSCLC	NR	1 year	Tac	Yes	-
Braun et al., 2020 [61]	Case report (1)	Nivolumab	NSCLC	1 cycle	3 years	Tac	Yes	-
Owoyemi et al., 2020 [62]	Case series (8)	Nivolumab	HCC recurrence	0.9 months	NR	Sirolimus	Yes	-
		Nivolumab	HCC recurrence	1 cycle	NR	Tac	No	No
		Nivolumab	HCC recurrence	0.9 months	NR	Tac	No	No
		Nivolumab	HCC recurrence	2.7 months	NR	Sirolimus	No	No
		Pembrolizumab	Melanoma	1 month	NR	Tac, MMF, Steroid	Yes	Yes
		Pembrolizumab	Melanoma	8.4 months	NR	MMF, Everolimus	No	Yes
		Nivolumab	SCC	15.4 months	NR	Tac, MMF, Steroid	No	Yes
		Nivolumab	HCC recurrence	0.9 months	NR	Tac	No	No
Pandey et al., 2020 [63]	Case report (1)	Ipilimumab	HCC recurrence	27 months	7.5 years	Tac	No	Yes
Zhuang et al., 2020 [64]	Case report (1)	Nivolumab	HCC recurrence	12 cycles	2 years	Tac	No	No
Ben Khaled et al., 2021 [65]	Case report (1)	Atezolizumab + Bevacizumab	HCC recurrence	9 months	4 years	NR	No	No
Bittner et al., 2021 [66]	Case report (1)	Nivolumab	PTLD	14 months	11 years	MMF	Yes	Yes
Brumfiel et al., 2021 [67]	Case report (1)	Nivolumab	SCC	15 months	21 years	Tac, MMF, Steroid	No	Yes
Shi et al., 2021 [68]	Case series (5)	Toripalimab	ICC recurrence (1 case)	1–6 months	NR	Sirolimus/Everolimus	No	Yes (3 cases) No (2 cases)
			HCC recurrence (4 cases)					
Tsung et al., 2021 [69]	Case report (1)	Cemiplimab	SCC	NR	NR	Tac	No	Yes
Kondo et al., 2022 [70]	Case report (1)	Nivolumab	SCC	4 cycles	3 years	MMF, Cyclosporine	No	No
Yang et al., 2022 [71]	Case series (2)	Atezolizumab + Bevacizumab	HCC recurrence	7 cycles	NR	NR	No	Yes
			HCC recurrence	2 cycles	NR	NR	No	No
Di Marco et al., 2023 [72]	Case series (5)	Nivolumab (1 case)	HCC recurrence	NR	1.2 years	Tac/Sirolimus/Everolimus	No	No
		Nivolumab + Bevacizumab (4 cases)					No (3 cases) Yes (1 case)	Yes (2 cases) No (2 cases)
Rudolph et al., 2023 [35]	Case series (4)	Atezolizumab + Bevacizumab	HCC recurrence	7 cycles	2 years	Tac, MMF, Steroid	No	No
		Atezolizumab + Bevacizumab	HCC recurrence	4 cycles	0.8 years	Tac, MMF, Steroid	No	No
		Nivolumab	Small bowel adenocarcinoma	1 cycle	4.2 years	Tac, Steroid	Yes	-
		Nivolumab	SCC	1 cycle	10 years	MMF, Sirolimus	No	No

Abbreviations: CRC, colorectal cancer; HCC, hepatocellular carcinoma; ICC, intrahepatic cholangiocarcinoma; ICI, immune checkpoint inhibitor; IS, immunosuppression; PTLD, post-transplant lymphoproliferative disorder; MMF, mycophenolate mofetil; NR, not reported; NSCLC, non-small-cell lung cancer; LT, liver transplantation; SCC, squamous cell carcinoma; Tac, Tacrolimus; Thymo, Thymoglobulin.

**Table 4:** Timings and patterns of HCC recurrence in studies on ICIs after LT.

Study	Time from LT to Recurrence (Months)	Recurrence Site
De Toni et al., 2017 [45]	11	Liver, adrenal gland, mesentery
Friend et al., 2017 [47]	36	Lung
	12	Lung
Varkaris et al., 2017 [48]	72	Retroperitoneal lymph nodes
Gassmann et al., 2018 [51]	24	Lung, retroperitoneal lymph nodes
Nasr et al., 2018 [53]	48	Lung
Rammohan et al., 2018 [54]	40	Lung
Al Jarroudi et al., 2020 [58]	33	Liver
	12	Adrenal gland, lung, mediastinal lymph nodes
	24	Liver, lung
Anugwom et al., 2020 [60]	12	Lung
Owoyemi et al., 2020 [62]	8	NR
	12	NR
	25	NR
	42	NR
	8	NR
Pandey et al., 2020 [63]	67	Liver
Zhuang et al., 2020 [64]	6	Lung
Ben Khaled et al., 2021 [65]	48	Liver, lung, retroperitoneal lymph nodes
Shi et al., 2021 [68]	16	Liver
	9	Lung
	14	Liver, lung, peritoneum
	12	Liver
	11	Liver, peritoneum
Yang et al., 2022 [71]	NR	Lung, sacral spine
	8	Liver, lung, peritoneum
Rudolph et al., 2023 [35]	24	NR
	6	NR

Abbreviations: LT, liver transplantation; NR, not reported.
